# Effects of antibiotics on chicken gut microbiota: community alterations and pathogen identification

**DOI:** 10.3389/fmicb.2025.1562510

**Published:** 2025-04-30

**Authors:** Ruiqi Zhan, Yining Lu, Yuan Xu, Xiaokun Li, Xilong Wang, Guanliu Yu

**Affiliations:** College of Life Sciences, Shandong Normal University, Jinan, China

**Keywords:** antibiotics, chicken, gut microbiota, single-molecule real-time sequencing, bacteria

## Abstract

The extensive use of antibiotics in animal husbandry, either for therapeutic purposes or as growth promoters, has raised significant concerns about their effects on poultry. However, when antibiotics are used as therapeutic agents, their impact on the gut microbiota of poultry remains unknown. This study aimed to address this gap by simulating therapeutic application of six frequently used antibiotics (lincomycin hydrochloride, gentamicin sulfate, florfenicol injection, benzylpenicillin potassium, ceftiofur sodium, and enrofloxacin infection) and investigated their effects on the composition and structure of poultry gut microbiota. Single-molecule real-time 16S rRNA sequencing was performed to analyze fecal samples collected from chickens treated with each antibiotic to assess the impact of antibiotic exposure on gut community diversity and dominant microbial species. Although the results demonstrated that antibiotic exposure reduced gut microbiota diversity and disrupted community stability, the impacts of different antibiotics differed considerably, specifically in the number of ASVs. Notably, the dominant bacterial phyla—*Pseudomonadota* and *Bacillota—*was largely consistent across different antibiotic exposures, except 11 days after gentamicin sulfate exposure. Moreover, six third-category pathogens were identified in fecal samples, namely, *Shigella boydii*, *Escherichia coli*, *Shigella flexneri*, *Salmonella enterica*, *Corynebacterium bovis*, *Proteus mirabilis*. Of these, three strains of *Corynebacterium bovis* were identified as potential novel pathogenic bacteria. These findings demonstrate the critical importance of rational antibiotics use in animal husbandry. This study provides a scientific basis for improving current antibiotics use in the treatment and prevention of poultry diseases, advancing the standardization and precision of antibiotic usage.

## 1 Introduction

Poultry is a globally important source of meat and dietary protein (Whitton et al., 2021). However, the poultry industry is significantly affected by disease outbreaks, which cause substantial economic losses for farmers (Muhammad et al., 2020). Antibiotics are extensively used to mitigate these losses (Lhermie et al., 2022), they are administered in avian husbandry for either therapeutic objectives or as stimulants for growth enhancement. Antibiotic growth promoters operate via conventional anti-inflammatory mechanisms and potentially stimulate poultry growth and optimize feed conversion efficiency by modulating mitochondrial activity and gut microbiota (Fernández Miyakawa et al., 2024). However, excessive use disrupts gut microbiota, impairs gut barrier function, and weakens immune responses, increasing susceptibility to infections (Guo et al., 2021).

The gut microbiota is vital in drug metabolism, affecting both drug efficacy and toxicity by regulating host metabolic pathways and competition for exogenous substrates. Therapeutic drugs, including antibiotics, can inadvertently disrupt gut microbiome, causing imbalances in microbial communities, which has potential long-term consequences (Wilson and Nicholson, 2016). Antibiotics, specifically, reduce gut microbiota diversity, disrupt metabolic functions, and increase the risk of antibiotic-induced diarrhea and recurrent *Clostridium difficile* infections (Mullish and Williams, 2018; Lesniak et al., 2021). For instance, ciprofloxacin administration can significantly reduce gut microbial richness and diversity in 3–4 days (Dethlefsen and Relman, 2011). Furthermore, antibiotics in feed exert site-specific effects on gut microbiota composition (Looft et al., 2014). Fecal samples are frequently employed to evaluate gut microbiota composition (Suchodolski et al., 2005) and its correlation with animal growth (Wang et al., 2021).

Recent advancements in gene sequencing, especially high-throughput sequencing, have facilitated detailed analyses of microbial communities. Although second-generation sequencing technologies, such as Illumina, are widely used, they are limited by short read lengths and lower accuracy (Bi et al., 2024). Single-molecule real-time (SMRT) sequencing, a third-generation technology developed by PacBio, offers superior read lengths and accuracy (Glenn, 2011; Wei and Zhang, 2018). Several studies have investigated the SMRT technique. For example, SMRT technology was used to sequence the complete genome of *Escherichia coli E28* (Imke et al., 2017) and analyze the diversity of intestinal mucosa microbiota (Zhang C. Y. et al., 2021; Zhang T. et al., 2021). SMRT sequencing improves accuracy through circular consensus sequencing. Normally, SMRT sequencing directly analyzes extracted DNA without requiring amplification by polymerase chain reaction (PCR). But we employed PCR to specifically amplify the full-length 16S rRNA gene. This amplification step was used to ensure sufficient template quantity for targeted sequencing, and it does not contradict the inherent advantages of SMRT technology.

This study investigated the effects of various antibiotics on chicken fecal microbiota using SMRT sequencing. We performed diversity, species composition and phylogenetic analyses of SMRT sequencing results based on the hypothesis that antibiotics destroy chicken gut microbes to determine the specific manifestations of the effects of antibiotics on chicken gut microbes. Furthermore, we identified the affected key strains based on changes in gut microbial composition under antibiotic exposure, and provided insights that facilitate the development of precision medicine strategies to reduce disease spread, optimize feed utilization, and lower production costs. Ultimately, this study would provide a scientific basis for rational antibiotic use in poultry farming, minimize antibiotic residues in food, and ensure adherence to food safety standards.

## 2 Materials and methods

### 2.1 Experimental design

The study was conducted at Shandong Normal University in September 2024. Forty-nine 3-day-old, specific-pathogen-free chickens were purchased from Spirax Ferrer Poultry Science and Technology Co., Ltd., Jinan, China and underwent a 5-day acclimatization period in the experimental environment. The 12-day trial provided all chickens with ad libitum access to food and water. Windows were opened during the day and partially closed at night to regulate temperature and ventilation, ensuring a comfortable rearing environment.

After acclimatization, the chickens were randomly divided into seven groups: normal control (NC), lincomycin hydrochloride (LH), gentamicin sulfate (GS), florfenicol injection (FI), benzylpenicillin potassium (BP), ceftiofur sodium (CS) and enrofloxacin infection (EI). To fulfill the critical requirement for replication, we assigned seven chickens to each experimental group—each antibiotic treatment group had seven replicates and each replicate had one chicken. This design ensured statistical robustness and reliability in identifying the effects of antibiotic treatment. Each group was administered a different antibiotic via intramuscular injection, with dosages and treatment durations adhering to manufacturer’s instructions. The specific group assignments and corresponding antibiotic treatments are detailed in Supplementary Table S1.

Fecal trays were replaced daily, and fresh fecal samples (0.2–0.5 g) were collected at multiple time points, namely, at days 0 (day of antibiotic treatment), 3, 7, and 11 after treatment. Because the seven chickens in each group were housed separately, we randomly collected three samples from fecal trays for sequencing, each sample weighed approximately 5 grams. Foreign materials, such as feathers and feed particles, were removed from the collected fecal samples. The samples were split into two equal parts: one for DNA extraction and the other stored at −80°C until subsequent analysis. These procedures were meticulously standardized to ensure consistent and reliable sample handling across all experimental groups and time points. All animal experiments complied with the guidelines of the Shandong Animal Ethics Committee and relevant biosecurity regulations. The protocol received approval from the Animal Care and Use Committee of Shandong Normal University (AEECSDNU2024126).

### 2.2 DNA extraction

DNA extraction utilized the MagBeads FastDNA Kit (116564384C1) as per the manufacturer’s protocol (MP Biomedicals, Solon, CA, United States). Extracted DNA quality was assessed via 0.8% agarose gel electrophoresis, and the concentration was determined by a NanoDrop NC-1000 spectrophotometer (Thermo Fisher Scientific, Waltham, MA, USA). DNA samples meeting quality criteria were stored at −80°C for subsequent library preparation.

### 2.3 PCR amplification and SMRT sequencing

Full-length 16S rRNA (~ 1,500 bp) was amplified by PCR to analyze bacterial diversity, using forward primer 27F (5′–barcode +AGAGTTTGATCMTGGCTCAG–3′) and reverse primer 1492R (5′–ACCTTGTTACGACTT–3′). The amplification process included an initial denaturation step at 98°C for 5 min, followed by 25–30 cycles of denaturation (98°C, 30 s), annealing (56°C, 30 s), extension (72°C, 45 s), and a final extension at 72°C for 10 min. The PCR products were purified using Agencourt AMPure Beads (Beckman Coulter, Indianapolis, IN, USA) and quantified with the Quant-iT PicoGreen dsDNA Assay Kit (Invitrogen, Carlsbad, CA, United States) following the manufacturers’ instructions. The 16S rRNA amplicons were sequenced on the PacBio Sequel II platform with the help of Personalbio Biotechnology Co., Ltd. Raw sequence data were deposited in the NCBI database (accession number PRJNA1194745).

### 2.4 Microbiota community diversity analysis

The raw sequences from SMRT sequencing were processed on the Personalbio Cloud platform. Sequences that did not match the primers were discarded using QIIME2 (version 2022.11). The DADA2 plugin was used for denoising, quality filtering, dereplication and chimera removal (Callahan et al., 2016). High-quality reads were aligned and clustered into nonsingleton amplicon sequence variants (ASVs) via MAFFT (Katoh et al., 2002). The relative abundance of each sample was calculated using ASV rarefaction.

Alpha diversity indices, such as the Simpson diversity index, Shannon diversity index, and Chao1 richness estimator, were derived from ASV table generated by QIIME2. Structural variations in the gut microbiota across groups were analyzed with principal coordinate analysis (PCoA) (Anderson and Willis, 2003). A Venn diagram visualized ASVs differences among groups based on abundance data. QIIME2 was used to obtain the composition and abundance tables at the phylum, family, genus, and species levels, and the results were presented in bar charts. Bacterial ASVs with relative abundances exceeding 10.00% were considered dominant species.

### 2.5 Biomarker prediction

Biomarkers function as early warning signals reflecting gut microbiota changes, such as harmful bacterial proliferation. This enables breeders to detect issues early and implement preventive measures to curb disease spread.

LEfSe multilevel discriminant analysis was used to identify high-dimensional biomarkers and genomic features (Segata et al., 2011). In LEfSe, the nonparametric Kruskal–Wallis (KW) test assessed species abundance differences across groups, followed by the Wilcoxon rank-sum test to confirm subgroups consistency. Lastly, linear discriminant analysis (LDA) was used to determine the impact of these species on group differences.

### 2.6 Functional analyses

We analyzed the pathogenic potential and microbial consortial characteristics of bacterial communities across the antibiotic treatment groups to investigate their biological functions. Metabolic functional profiles were evaluated using PICRUSt2 (Douglas et al., 2020). Functional annotations, derived from 16S rRNA sequencing data, were obtained from the KEGG database^1^ using default system parameters^2^ (Langille et al., 2013).

### 2.7 Phylogenetic analysis of potential novel pathogenic bacterial strains

To identify potential pathogenic microorganisms for humans and poultry, we compared the top 50 bacterial species from this study with entries in the Catalogue of Pathogenic Microorganisms Infecting Humans (Vision 2021, P.R. China Ministry of Health; Song et al., 2023; Chen et al., 2024).

We then analyzed the full-length 16S rRNA sequences of the pathogenic bacteria obtained by SMRT sequencing and performed detailed phylogenetic analyses. The ASV sequences of each pathogenic bacterium were compared with representative sequences from the NCBI database using the ClustalW function in the MegAlian program (DNAStar software). Sequences with gene alignment < 96% were screened. Phylogenetic trees were constructed using the neighbor-joining method within the MEGA program (Tamura et al., 2011). The genetic sequences of novel pathogenic bacteria were identified based on two criteria: (1) the strains’ sequences were located on distinct branches of the phylogenetic tree, and (2) their nucleic acid similarity rates were below 97.0% (Chen et al., 2024; Song et al., 2023, 2024). The gene sequences of these novel bacterial strains identified in this study were uploaded to the GenBank database (accession numbers PQ676072, PQ676073, PQ676074).

### 2.8 Statistical analysis

Relative abundance values were expressed as mean ± standard deviation. One-way analysis of variance and multiple comparison tests were conducted by SAS 9.1 software (SAS Institute, Inc., Cary, NC, United States) to assess group differences. Statistical significance was set at *p*-value < 0.05 or < 0.01.

## 3 Results

### 3.1 Bacterial diversity in fecal samples

**Figure 1:**
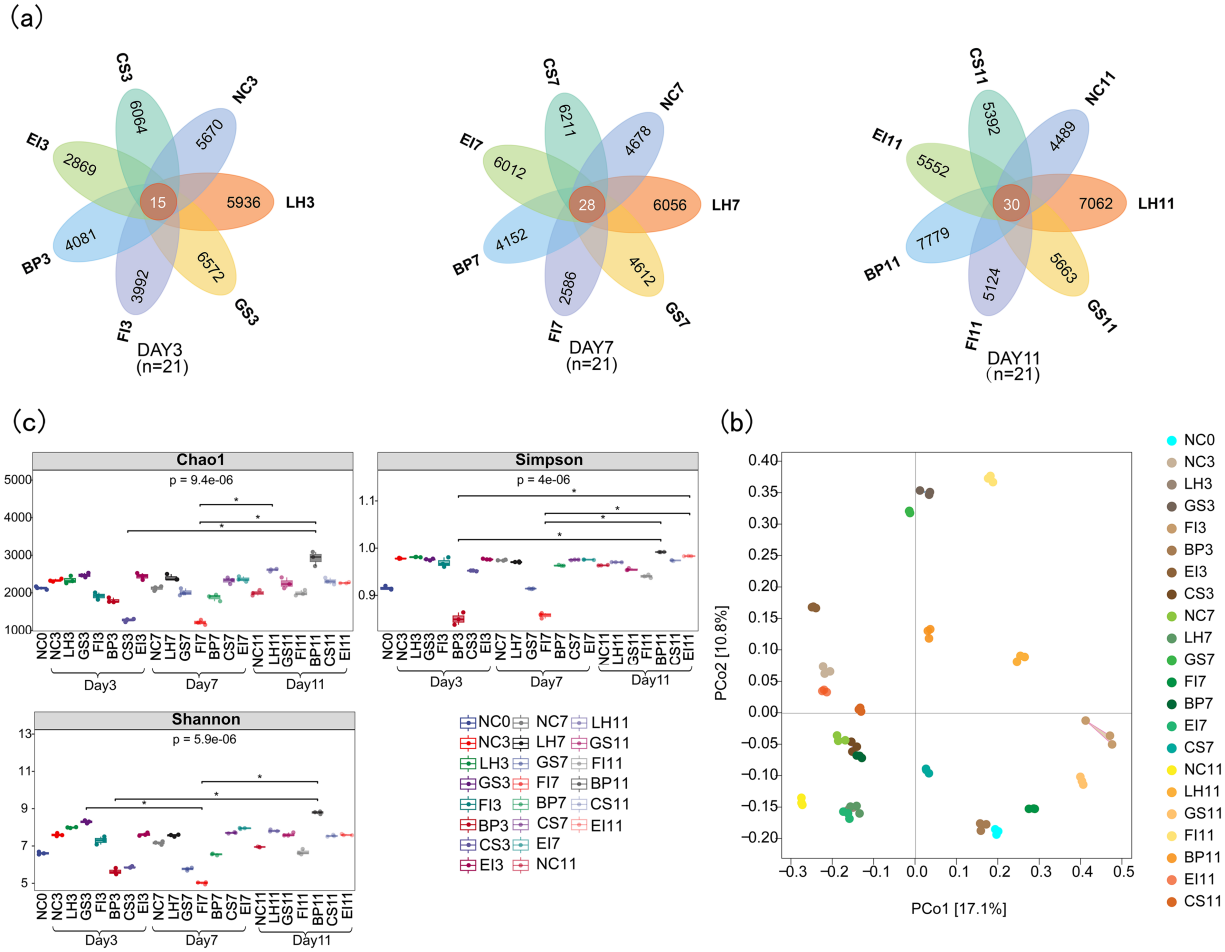
Venn diagram of amplicon sequence variants **(a)**; principal coordinate analysis plots **(b)**; and Chao1, Simpson, and Shannon indices **(c)** of bacterial communities.

A mean number of 62,749 ASVs were identified from the total 66 samples, with a standard error of 229.2 (Supplementary Table S1). All antibiotics significantly influenced ASV counts, with notable variations across groups. On day 3, ASV counts for groups LH3, GS3, FI3, BP3, CS3, EI3, and NC3 were 5,936, 6,572, 3,992, 4,081, 2,869, 6,064, and 5,670, respectively, with 15 common ASVs. On day 7, ASV counts for these groups were 6,056, 4,612, 2,586, 4,152, 6,012, 6,211, and 4,678, respectively, with 28 common ASVs. On day 11, ASV counts for the groups were 7,092, 5,663, 5,124, 7,779, 5,552, 5,392, and 4,489, respectively, with 30 common ASVs (Figure 1a).

PCoA based on the Bray–Curtis measure revealed that samples from the same group were more similar, whereas those from different groups were more dispersed. Thus, intragroup differences were smaller than intergroup differences. Intestinal flora components from different groups showed significant variation (*p* < 0.05), indicating effective group separation (Figure 1b).

Alpha diversity, which indicates the abundance and homogeneity of microbial communities, is often measured using the Chao1, Shannon, and Simpson indices. Box plots created based on the Kruskal–Wallis test results revealed significant differences in gut flora diversity under different antibiotic exposures (Chao1, *p* = 9.4e-06; Shannon, *p* = 4e−06; Simpson, *p* = 5.9e−06; Figure 1c). The Chao1, Shannon, and Simpson indices generally decreased and then increased with prolonged antibiotic exposure. For instance, the Simpson index on day 3 after BP exposure was significantly lower than on day 11 (*p* < 0.05). These results indicate that antibiotic exposure reduces gut flora diversity, with varying impacts across different antibiotics.

### 3.2 Bacterial taxonomy and relative abundance in fecal samples

**Figure 2:**
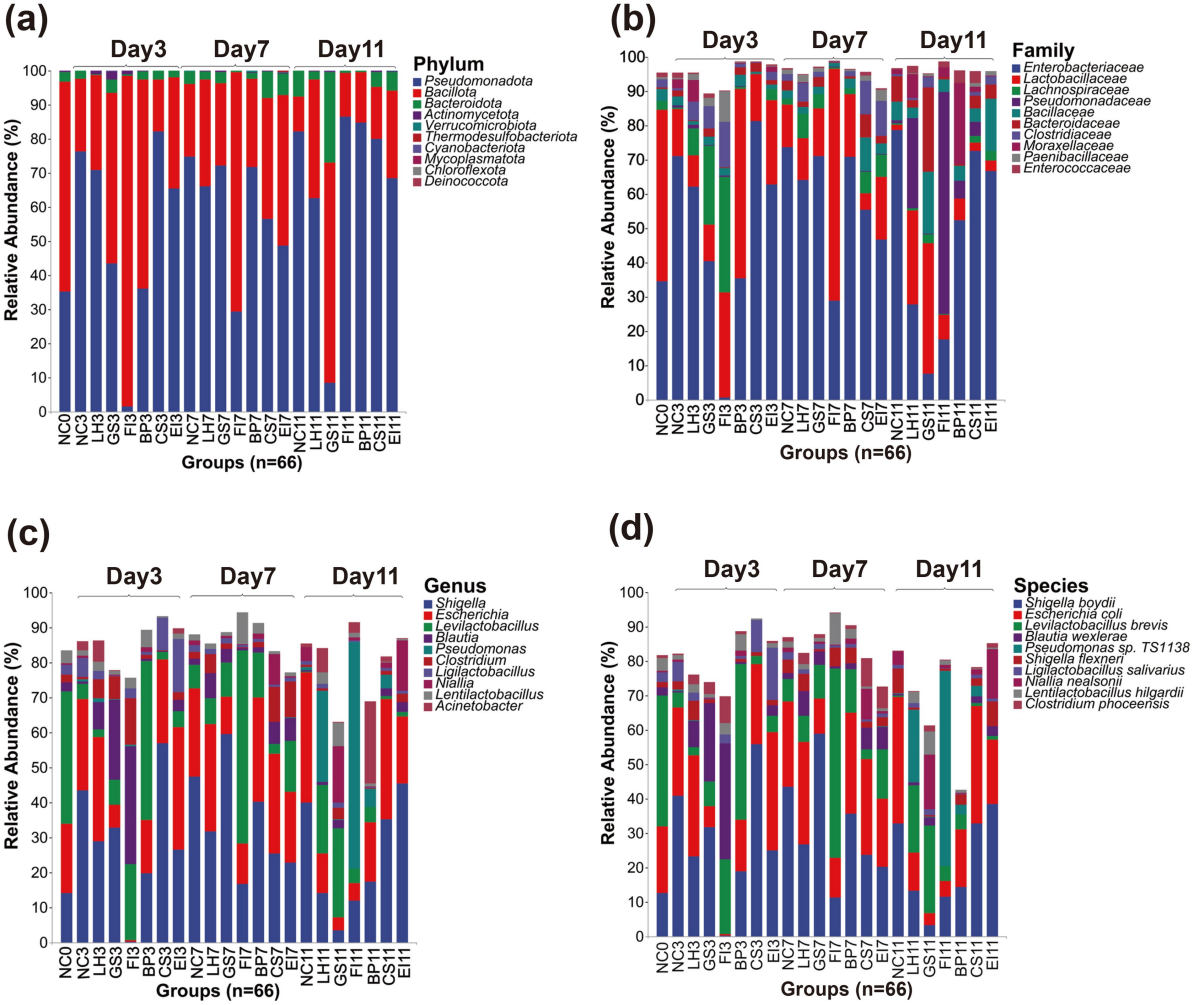
Amplicon sequence variants of the top 10 most relatively abundant bacteria detected at the phylum **(a)**, family **(b)**, genus **(c)**, and species **(d)** levels.

Relative abundance was analyzed at the phylum, family, genus, and species levels. *Pseudomonadota* (59.25% on average) and *Bacillota* (36.23%) were the dominant phyla. Notably, on day 3 after exposure to FI, *Pseudomonadota* decreased sharply (1.53%), whereas *Bacillus* significantly increased (96.85%). These two phyla showed comparable percentages with no significant differences as the dominant phyla in the NC and other treatment groups. These microbial abundance changes in the FI group gradually recovered with exposure time and returned to normal levels by day 11. In contrast, an abnormality was noted in the GS group until day 11, when *Bacteroidota* spp. were detected (26.41%). Furthermore, the percentage of *Pseudomonadota* was significantly lower than those in the NC group (8.44% versus 82.25%, *p* < 0.05), which was not found in the other groups (Figure 2a). At the phylum level, the LH, BP, CS, and EI groups did not show much difference compared to the NC group.

The dominant families were *Enterobacteriaceae* (51.04% on average) and *Lactobacillaceae* (20.04%). In contrast, on day 3, the percentage of *Enterobacteriaceae* in the FI group was significantly lower than that in the NC group (0.64% vs. 71.15%, *p* < 0.05), whereas that of *Lachnospiraceae* (33.80%) was higher than that in the NC group. On day 7, the *Lachnospiraceae* in the FI group significantly decreased, reaching a level similar to that of the NC group. However, on day 11, a notably high percentage of *Pseudomonadaceae* (64.81%) was noted, which was not observed in the other groups. The percentages in the LH group were largely similar to those of the NC group on days 3 and 7. However, on day 11, *Pseudomonadaceae* (26.27%) spp. were detected at a percentage similar to that of the FI group. *Lachnospiraceae* (23.07%) spp. were also observed in the GS group on day 3 at a percentage similar to those of other groups on day 7. However, the percentage of *Enterobacteriaceae* was lower than that of the NC group on day 11 (7.50% vs. 78.66%, *p* < 0.05). None of the EI, BP, and CS exposed from 3 to 11 days showed significant differences, except the NC group (Figure 2b).

*Shigella* (28.84% on average), *Escherichia* (20.05%), *Levilactobacillus* (13.19%) were the dominant genera. The microbial percentages in the CS and EI treatment groups did not differ significantly from those in the control group. On day 3, the FI group showed an extremely abnormal situation compared with the other groups. Specifically, *Shigella* was significantly lower than that in the NC group (0.21% vs. 56.88%, *p* < 0.05), and *Blautia* (33.71%) and *Levilactobacillus* (21.68%) were the dominant genera. On day 7, the percentage of *Levilactobacillus* continued to increase until it was no longer dominant on day 11. However, the microbial percentages in the FI group did not return to normal levels, and *Pseudomonas* (64.76%) spp. were even detected. Furthermore, the BP group was dominated by *Levilactobacillus* with a significantly higher percentage than that in the NC group (45.32% versus 4.37%, *p* < 0.05) on day 3. However, the difference gradually diminished by day 7. The effect of LH treatment was indicated by the presence of *Pseudomonas* (26.22%) on day 11 and *Levilactobacillus* (19.53%) as dominant genera relative to those in the control group (Figure 2c).

*Shigella boydii* (26.11% on average), *Escherichia coli* (19.67%), *Levilactobacillus brevis* (13.19%) were the most dominant species. However, prolonged exposure to antibiotics led to changes in the dominant strains of the intestinal tract, which were very similar to those at the genus level. On day 3, the FI group was dominated by *Blautia wexlerae* (33.70%) and *Levilactobacillus brevis* (21.68%), with the percentage of *S. boydii* being much lower than that in the NC group (0.19% vs. 40.83%, *p* < 0.05). Furthermore, the microbial percentage levels did not recover by day 11. The CS and EI groups differed slightly in the percentage of *S. boydii* and *E. coli* as dominant strains on day 7; however, this difference was quickly offset. The BP group showed high levels of *Levilactobacillus brevis* (45.32%) on day 3, which was not observed in the other groups. Similarly, the GS group exhibited high levels of *Blautia wexlerae* (22.93%) on day 3, which was similar only to the FI group. Furthermore, although the composition recovered slightly on day 7, significant differences with the other groups were observed again on day 11, with *S. boydii* and *E. coli being* lower than those of the NC group (3.18 vs. 32.8, 3.53% vs. 36.81%, *p* < 0.05) (Figure 2d).

### 3.3 Biomarker prediction for fecal samples

**Figure 3:**
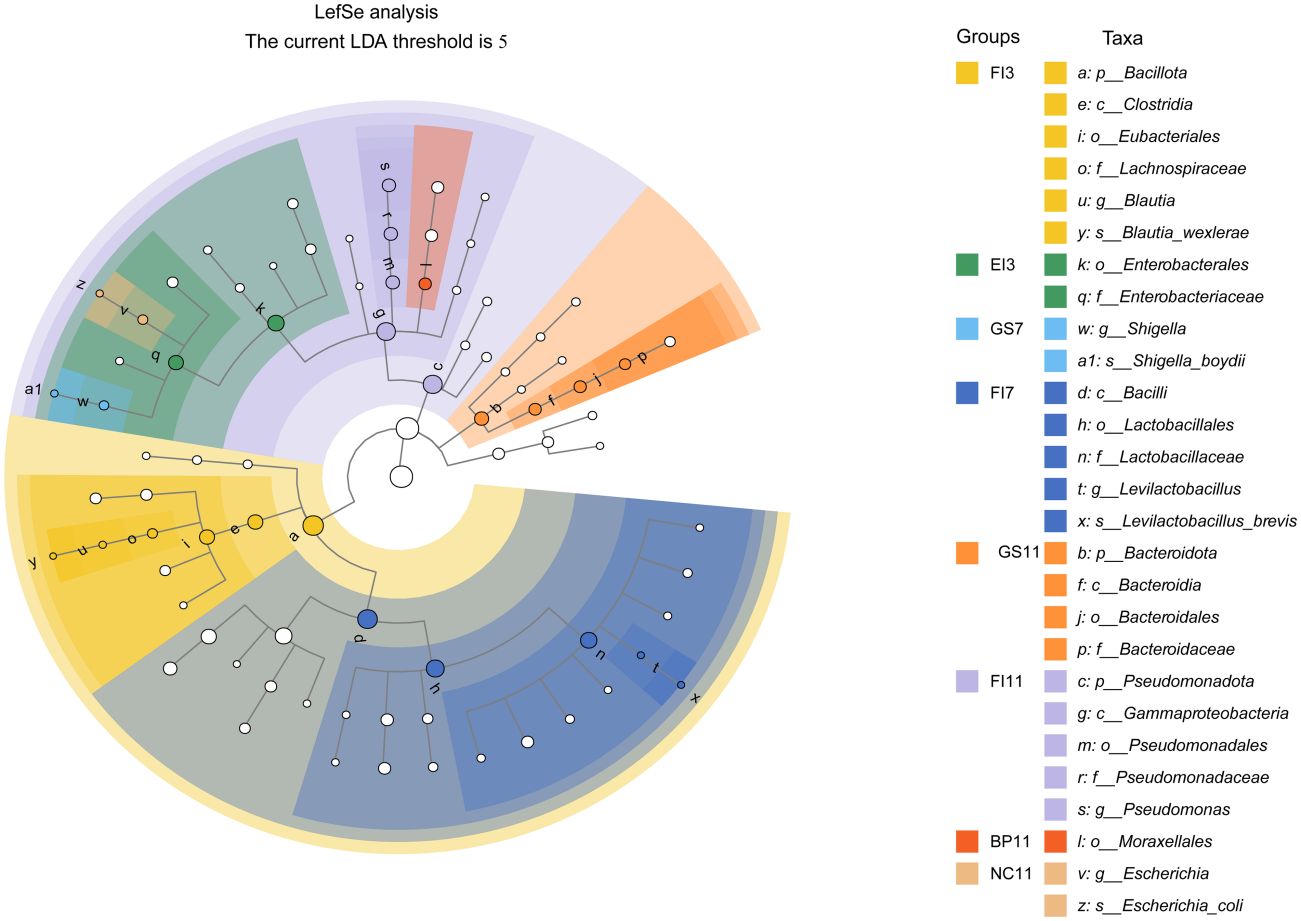
Linear discriminant analysis (LDA) effect size (LEfSe) of fecal samples collected from the various treatment groups. The cladograms indicate the results for bacteria. Solid dots of different colors indicate significant discriminative taxonomic nodes in the samples, while hollow dots represent non-discriminative taxonomic nodes. The branches are shaded based on the highest-ranked variety of each taxon (LDA = 5, *p* < 0.05).

LEfSe analysis was performed to identify statistically significant gut flora biomarkers across 22 groups, using an LDA threshold score of 5.0 to highlight differences in bacterial community structure. The relative abundance of most bacterial taxa among the above groups differed. In total, 27 intestinal microbial species showed significant differences in abundance among groups (*p* < 0.05). Potential biomarkers for the FI group included *p_Bacillota*, *c_Clostridia*, *o_Eubacteriales*, *c_Gammaproteobacteria*, *p_Pseudomonadota*, *c_Bacilli*, *o_Lactobacillales*, and *f_Lactobacillaceae*. For the GS group, potential biomarkers included *s_Shigella_boydii*, *g_Shigella*, *p_Bacteroidota*, and *c_Bacteroidia.* Biomarkers identified in the EI, BP, and NC groups were *o_Enterobacteriaceae*, and *f_Enterobacteriaceae*, *o_Moraxellales*, *g_Escherichia*, and *s_Escherichia_coli*, respectively (Figure 3).

These biomarkers provide insights into the effects of antibiotics on gut microbes and enhance our understanding of their growth dynamics.

### 3.4 Metabolic functions and pathways

**Figure 4:**
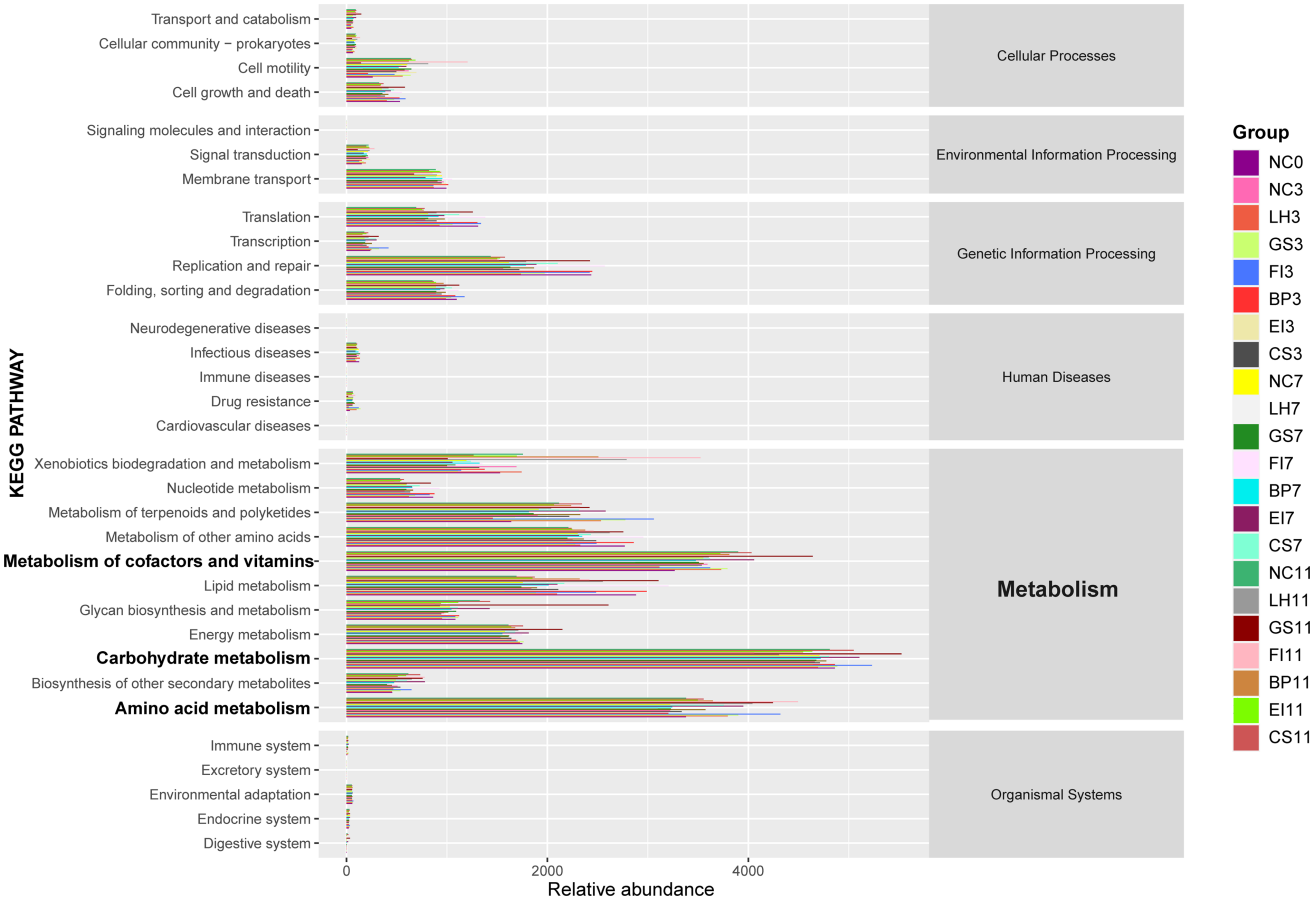
Metabolic functional profiles of bacteria across different groups, as determined by PICRUSt2 analysis.

Analysis of the intestinal microbiota pathways identified carbohydrate metabolism as the most abundant pathway (Figure 4). These pathways promote the growth and reproduction of specific strains, thereby supporting microbial homeostasis.

Furthermore, the abundant pathways for the metabolism of amino acids, cofactors, and vitamins indicate that intestinal microorganisms respond positively to antibiotic-induced environmental changes, exhibiting sustained normal physiological functions. Gut microbes modify their metabolic pathways and physiological functions based on the type and concentration of amino acids in the environment. Some vitamins and their derivatives act as signaling molecules, regulating microbial gene expression and enhancing microbial adaptation to environmental changes.

Therefore, gut microbiota modulate the metabolism of carbohydrates, amino acids, cofactors, and vitamins in response to antibiotic-induced environmental changes, thereby preserving microbial homeostasis and normal physiological functions.

### 3.5 Third category of pathogens

Among the top 50 most abundant bacterial species, six (*Shigella boydii*, *E. coli*, *Shigella flexneri*, *Salmonella enterica*, *Corynebacterium bovis*, and *Proteus mirabilis*) were listed in the Catalogue of Pathogenic Infecting Humans. These six species are classified as third-category pathogens.

### 3.6 Potential novel pathogenic bacterial strains

**Figure 5:**
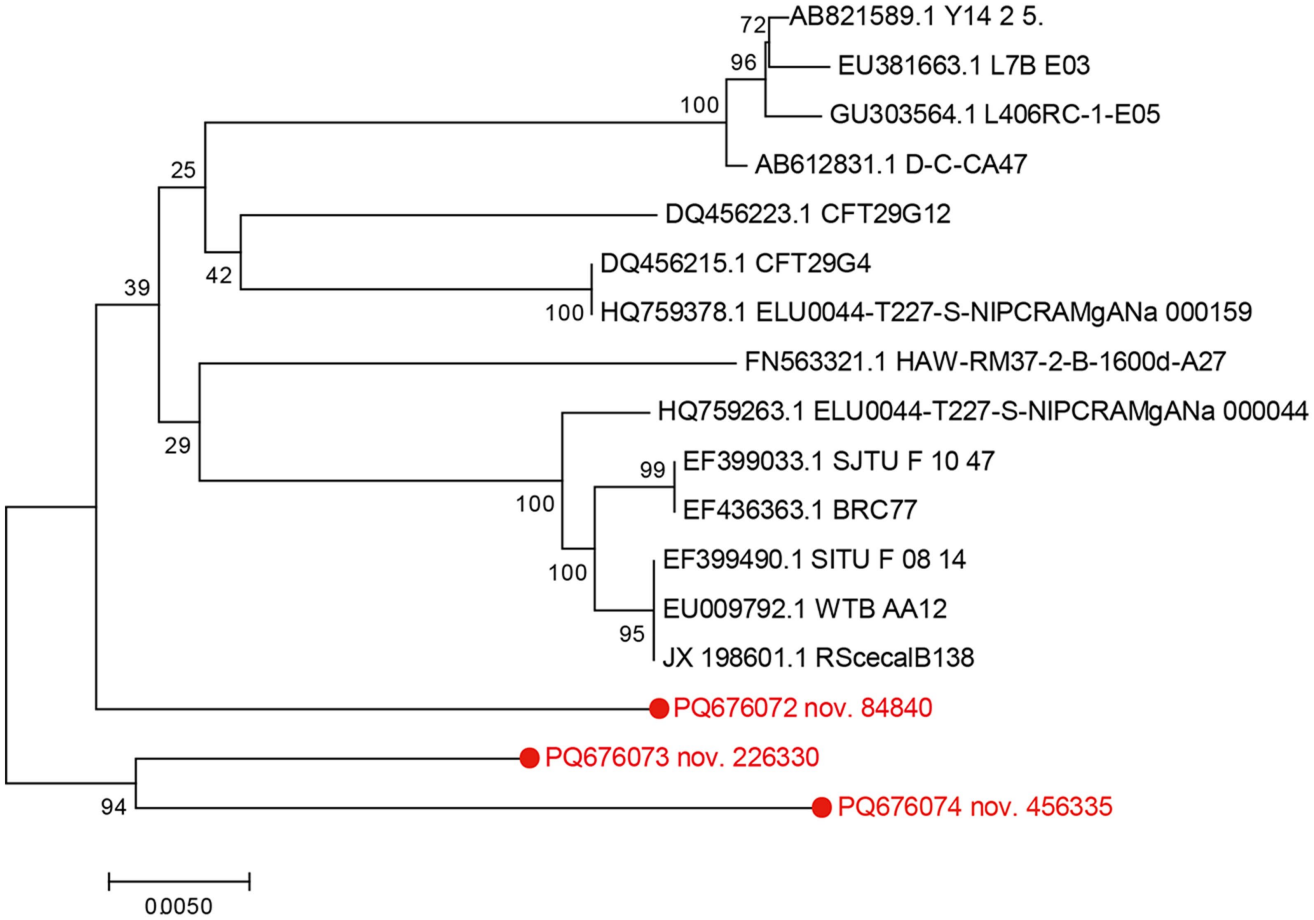
Phylogenetic tree based on 16S rRNA of potential novel *Corynebacterium bovis* strains in conjunction with reference strains. The genetic sequences of the four newly identified *Corynebacterium bovis* strains are indicated in red font. Constructed using MEGA 7.0 software using the neighbor-joining analysis, the phylogenetic tree presents 1,000 bootstrap replicates for confidence assessment. The scale bar indicates the number of nucleotide substitutions per site.

Table 1 highlights three potential novel *C. bovis* strains identified in fecal samples from the GS3, GS7, FI3, and CS11 groups. These novel strains showed < 96.0% nucleic acid identity with the 16S rRNA sequences in the GenBank database. The 16S rRNA genomes of these three novel bacteria strains shared 94.4–96.4% nucleic acid similarity with representative strains (Table 2). Moreover, a phylogenetic tree constructed via the neighbor-joining method revealed that the three novel *C. bovis* strains occupied distinct branches from other representative strains (Figure 5). The above results signify that these three *C. bovis* may be three novel pathogenic bacteria.

**Table 1:** Potential novel *Corynebacterium bovis* strains identified from fecal samples.

Bacteria name (GenBank No.)	No. of strains	Group distribution
*Corynebacterium* sp. ASV 84840 (PQ676072)	48	GS3, FI3, CS11
*Corynebacterium* sp. ASV 226330 (PQ676073)	32	GS3, GS7, CS11
*Corynebacterium* sp. ASV 456335 (PQ676074)	6	GS3

**Table 2:** Comparison of sequence similarity between the potentially novel *Corynebacterium bovis* strains identified in this study and other representative strains.

Divergence (%)		Nucleotide identity (%)																
		1	2	3	4	5	6	7	8	9	10	11	12	13	14	15	16	17
1	**PQ676072**	***	96.2	94.5	**95.6**	**96.1**	**95.4**	**95.4**	**96.1**	**96.1**	**96.1**	**96.0**	**96.0**	**96.0**	**95.6**	**96.1**	**95.6**	**95.6**
2	**PQ676073**	3.9	***	96.2	**95.4**	**96.0**	**95.2**	**95.3**	**95.6**	**95.6**	**95.6**	**95.6**	**95.6**	**95.5**	**95.6**	**96.4**	**95.7**	**95.7**
3	**PQ676074**	5.7	3.9	***	**95.3**	**94.9**	**95.1**	**95.1**	**94.8**	**94.8**	**94.8**	**94.7**	**94.7**	**94.6**	**95.4**	**95.1**	**94.4**	**94.4**
4	AB821589	4.6	4.7	4.9	***	95.5	99.5	99.7	96.0	96.0	96.0	96.0	96.0	95.9	99.7	96.3	96.7	96.7
5	FN563321	4.0	4.1	5.3	4.7	***	95.3	95.3	96.6	96.6	96.6	96.5	96.5	96.6	95.7	96.7	95.8	95.8
6	GU303564	4.7	4.8	5.0	0.4	4.7	***	99.4	95.7	95.7	95.7	95.7	95.7	95.7	99.4	96.1	96.5	96.5
7	EU381663	4.7	4.9	5.0	0.3	4.8	0.4	***	95.9	95.9	95.9	95.8	95.8	95.8	99.6	96.1	96.6	96.6
8	JX198601	4.1	4.5	5.4	4.2	3.5	4.3	4.3	***	100.0	100.0	99.5	99.5	99.4	96.2	96.3	96.9	96.9
9	EF399490	4.1	4.5	5.4	4.2	3.5	4.3	4.3	0.0	***	100.0	99.5	99.5	99.4	96.2	96.3	96.9	96.9
10	EU009792	4.1	4.5	5.4	4.2	3.5	4.3	4.3	0.0	0.0	***	99.5	99.5	99.4	96.2	96.3	96.9	96.9
11	EF436363	4.1	4.6	5.5	4.2	3.5	4.3	4.3	0.5	0.5	0.5	***	100.0	99.3	96.2	96.2	96.8	96.8
12	EF399033	4.1	4.6	5.5	4.2	3.5	4.3	4.3	0.5	0.5	0.5	0.0	***	99.3	96.2	96.2	96.8	96.8
13	HQ759263	4.1	4.6	5.6	4.2	3.4	4.3	4.3	0.6	0.6	0.6	0.7	0.7	***	96.1	96.2	96.9	96.9
14	AB612831	4.5	4.5	4.8	0.3	4.4	0.5	0.4	4.0	4.0	4.0	4.0	4.0	4.0	***	96.5	96.7	96.7
15	DQ456223	4.1	3.7	5.1	3.8	3.4	3.9	4.0	3.8	3.8	3.8	3.9	3.9	3.9	3.5	***	97.2	97.2
16	HQ759378	4.5	4.4	5.9	3.3	4.4	3.5	3.5	3.2	3.2	3.2	3.3	3.3	3.2	3.3	2.8	***	100.0
17	DQ456215	4.5	4.4	5.9	3.3	4.4	3.5	3.5	3.2	3.2	3.2	3.3	3.3	3.2	3.3	2.8	0.0	***

Nucleotide sequences of three novel Corynebacterium bovis and 14 reference strains in bold.

## 4 Discussion

Poultry gut microbiota establishes early in life and remains relatively stable, albeit shaped by environmental and host factors (Hasan and Yang, 2019). Recent studies have demonstrated that antibiotic exposure causes significant shifts in microbiota composition and diversity (Zhang C. Y. et al., 2021; Zhang T. et al., 2021; Liang et al., 2023). However, large-scale studies with extensive data and large sample sizes on the impact of antibiotics on poultry gut microbiota remain scarce. This study addresses this gap by analyzing dynamic changes in poultry gut microbiota following exposure to six representative antibiotics using SMRT sequencing. This study highlights the distinct effects of different antibiotics on the gut microbiota, providing insights for the development of targeted strategies to reduce unnecessary antibiotic use and combat antibiotic resistance.

Antibiotic exposure impacts poultry gut microbiota in complex and multifaceted ways. Consistent with previous studies (Francino, 2016; Patangia et al., 2022), we found that antibiotics exposure significantly altered gut microbiota composition and diversity, which are key factors influencing microbiota stability. BP is a well-known antibiotic that was discovered by Alexander Fleming and has since revolutionized modern medicine by treating various bacterial infections. BP reduces host toxicity by inhibiting cell wall synthesis with minimal impact on human cells. BP and CS, both beta-lactam antibiotics, inhibit peptidoglycan synthesis by acylating transpeptidase to disrupt bacterial cell wall formation (Lima et al., 2020). Despite belonging to the same antibiotic class, their impacts on gut microbiota diversity differ. We found that microbiota diversity was significantly lower after BP exposure than after CS exposure. This could be due to the presence of beta-lactamase-producing *E. coli* and *Pseudomonas* sp. *TS1138* in the gut, which degrade or modify the antibiotic before it reaches the target site (Wilke et al., 2005). Compared with the more beta-lactamase-resistant CS, BP is more easily hydrolyzed, thereafter exhibiting reduced antibacterial efficacy in targeting specific sensitive bacteria. Furthermore, since BP primarily targets Gram-positive bacteria, it has a narrower antibacterial spectrum, causing a more uneven effect on gut microbiota and a greater reduction in diversity. A more diverse microbiota better maintains stability and more easily recovers from disturbances (Fassarella et al., 2021), indicating that BP exposure could lead to a more unstable microbiota community with reduced recovery potential. Taxonomic analysis further confirmed that by day 11, microbiota in chickens treated with EI and CS had largely recovered, whereas the parameters for those treated with BP still differed significantly from those of controls. These findings align with earlier studies indicating the lasting impact of BP on gut microbiota (Leclercq et al., 2017), although a mouse study reported that EI can also induce persistent microbiota changes (Elokil et al., 2020). Species differences, such as between mice and poultry, underscore the need for further studies on the recovery mechanisms of poultry microbiota after prolonged antibiotic exposure.

Bacillota and Bacteroidetes typically dominate chicken gut microbiota (Ye et al., 2021), However, after antibiotic exposure in this study, Pseudomonadota and Bacillota became predominant, except in the GS11 group. At the phylum level, Pseudomonadota and Bacillota collectively accounted for 95.48% of the average relative abundance across all samples. Therefore, antibiotic exposure may reduce Bacteroidetes colonization while increasing Pseudomonadota colonization in poultry gut. Increased Pseudomonadota abundance after antibiotic exposure may indicate gut microbiota dysbiosis, as their adaptive capabilities allow them to dominate microbial communities (Shin et al., 2015). A reduction in Bacteroidetes, which are associated with fat accumulation and improved growth performance in poultry (Chen et al., 2020), may indicate hindered growth.

While dominant bacterial phyla after antibiotic exposure were largely consistent across the different antibiotics tested, significant differences were observed at the family, genus, and species levels. The distinct changes induced in gut microbiota species composition also varied with antibiotic type. At 3, 7, and 11 days after GS exposure, *E. coli* abundance was significantly lower than that in the NC group. The reduction may be correlated with the pharmacological mechanism of GS, an aminoglycoside antibiotic extracted from *Micromonospora purpurea*, known for its broad-spectrum bacteriostatic effect. GS primarily targets Gram-negative bacteria, and, thus, strongly inhibits *E. coli* (Bai et al., 2022). GS binds to specific sites on the 16S rRNA of the *E. coli* 30S ribosomal subunit, disrupting mRNA translation. This causes incorrect tRNA pairing, abnormal amino acid incorporation, and protein synthesis disruption, ultimately killing the bacteria (Garneau-Tsodikova and Labby, 2016). FI is a protein synthesis inhibitor that exhibits bacteriostatic activity. It is a broad-spectrum antimicrobial of the chloramphenicol class with a similar mechanism of action as GS. FI is rapidly absorbed in the poultry gut (Watteyn et al., 2018). Its long half-life and good tissue distribution effectively treat respiratory and gastrointestinal infections, including salmonellosis and colibacillosis. The broad-spectrum antimicrobial properties of FI significantly reduced the abundances of *E. coli* and *S. boydii* in the FI group compared with those in the NC group. Specifically, FI binds tightly to the 50S subunit of the 70S ribosome (Guo et al., 2024), inhibiting peptidyl transferase and thereby impeding peptide chain elongation and formation, arresting protein synthesis. Reduced ethanol production by *E. coli*, which is linked to intestinal permeability, lowers gut oxygen levels (Dobrzanska et al., 2020), favoring the growth of anaerobic bacteria, such as *Clostridium phoceensis* and *L. brevis*, while suppressing aerobic bacteria, such as *S. flexneri* and *B. wexlerae*.

Our study has demonstrated that exposure to different antibiotics affects the diversity of gut microbial communities in poultry (Patangia et al., 2022). Moreover, because the gut microbiome is a complex microbial ecological network, the impacts of different antibiotics vary, (Fishbein et al., 2023), as established herein, further indicating variations in the exposure time required for different antibiotics to alter the community structure and in the recovery time of microbial communities. Specifically, CS and EI had the least impact on the composition of the chicken gut microbiota, caused the weakest disruption, and exhibited the fastest recovery times. By day 11, microbial levels had essentially returned to normal. In contrast, FI and GS exhibited the strongest destructive effects on the gut microbiota structure, which was evident from day 3 and persisted until day 11 without recovery. The LH group only showed significant differences compared with the NC group on day 11. Although BP disrupted the gut microbiota on day 3, the microbial levels had essentially recovered by day 7. Therefore, future studies need to further determine the specific times to reach maximum efficacy and for the efficacy to dissipate for each antibiotic.

Notably, this study identified three potentially novel *C. bovis* strains. The genome of *C. bovis* contains multiple copies of 16S rRNA gene (the rrnDB database). This genomic feature minimizes intragenomic sequence heterogeneity (i.e., sequence variations between multiple rRNA copies within the same genome), thereby reducing artifactual ASV (amplicon sequence variant) splitting caused by technical sequencing errors or natural copy number variations. Enhanced sequence uniformity improves taxonomic resolution at the strain level. For example, the sequencing *E. coli* 16S rRNA, based on the cyclic coherence sequencing technology of the PacBio platform, resulted in *E. coli* strains being accurately categorized into the O157:H7 and K12 subspecies branches, thereby demonstrating the utility of ASVs in accurately differentiating between strains (Callahan et al., 2019). Furthermore, soil bacterial analysis by sequence comparison of ASV numbers demonstrated that ASVs can represent strain species (Kim et al., 2024). Therefore, although the sequences of the three ASVs obtained showed 94–96% similarity to the published gene sequences of *C. bovis*, the SMRT sequencing results could be attributed to *C. bovis* based on their ASV numbers.

The sequences were obtained by SMRT 16S rRNA sequencing. Thus, not all potential novel pathogens may have been identified because of their relatively low abundance and the targeted nature of the sequencing, which targeted ribosomal genes rather than entire genomes (Chen et al., 2024). Identifying novel potential pathogens is vital for the prevention and control of poultry diseases. Future studies should include an in-depth analysis of poultry gut microbiota that particularly focuses on pathogenic microbial variants that emerge under antibiotic selective pressure.

One strategy for mitigating the impact of antibiotics is to coadminister other drugs or substances during antibiotic treatment to modulate antibiotic potency and minimize harm to beneficial bacteria. In particular, prebiotics can promote the growth of beneficial bacteria and help restore the microbiota structure and function (Bedu-Ferrari et al., 2022). Implementing these strategies may help preserve gut microbiota balance, support infection treatment, and mitigate the adverse effects of antibiotics. Future research should also focus on identifying relevant drugs or prebiotics and investigating the effectiveness and optimal applications of these strategies for more precise and less disruptive antibiotic treatment.

## 5 Conclusion

This study showed that antibiotics significantly affected microbial diversity and community structure, with FI, GS, and BP having the most significant and long-lasting effects. The CS and EI treatment groups showed significant early effects but faster recovery compared to the other groups. The LH treatment group showed late variability. Phylogenetic analysis revealed three potentially new pathogenic strains of *C. bovis*. Therefore, in developing a strategy for antibiotic use, antibiotics with the weakest impact on the gut flora and the fastest recovery, e.g., CS and EI, should be prioritized. The use of antibiotics significantly disruptive to the ecological balance of gut flora, e.g., FI, BP, and GS, should be minimized, considering therapeutic efficacy is ensured. Furthermore, antibiotic monotherapies should be avoided, and the combination of probiotics and antibiotic therapy should be considered to promote the growth of beneficial bacteria. This study further demonstrated the disruptive effects of certain antibiotics on the ecological balance of the poultry gut microbiota at specific doses, providing an important foundation for strategies to rationalize the use of antibiotics and mitigate resistance.

## Data Availability

The datasets presented in this study can be found in online repositories. The names of the repository/repositories and accession number(s) can be found in the article/Supplementary material.

## Footnotes

^1^ https://www.kegg.jp/

^2^ https://github.com/picrust/picrust2/wiki
