# Supplementary material for: Effects of antibiotics on chicken gut microbiota: community alterations and pathogen identification
Source: Front Microbiol. 2025 Apr 30;16:1562510. doi: 10.3389/fmicb.2025.1562510 (PMC12075146; doi:10.3389/fmicb.2025.1562510)
Supplement: Supplementary file 1 [file Data_Sheet_1.docx]

**
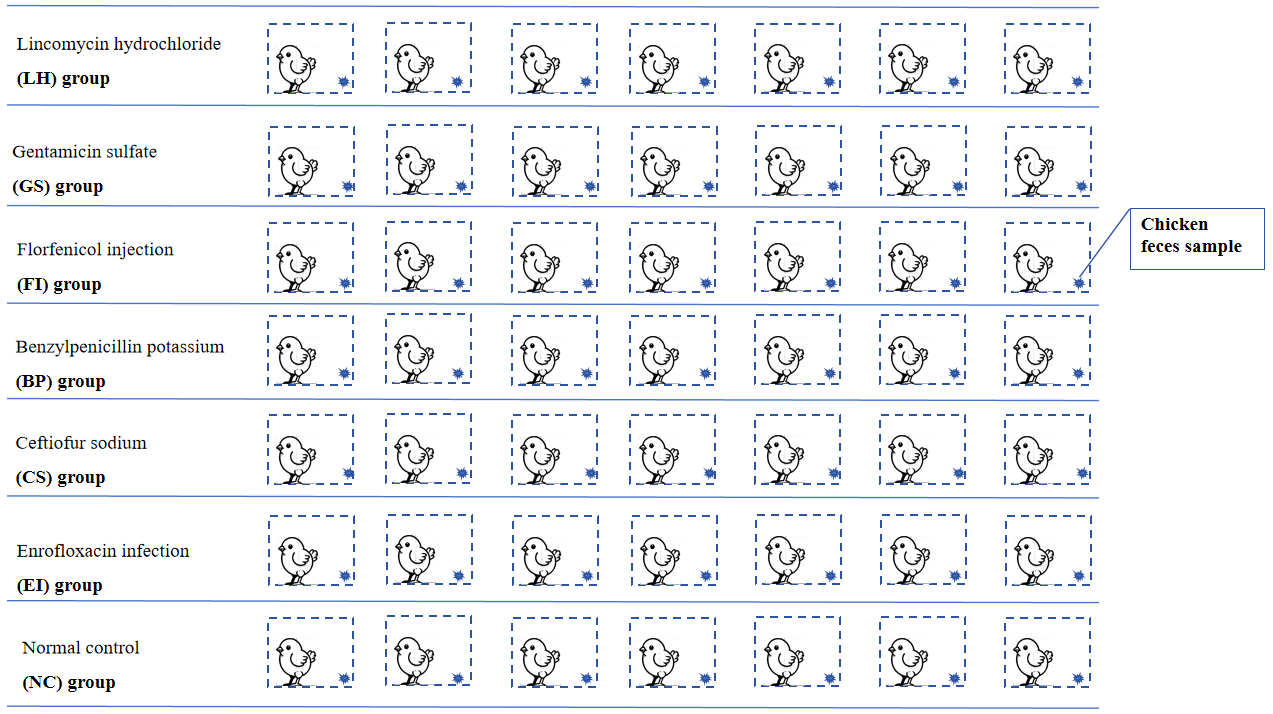
**

**Figure S1.** Schematic diagram of experimental grouping scheme: Forty-nine chickens were divided into 7 groups (NC, LH, GS, FI, BP, CS, EI), with 7 replicates in each group and each replicate has one chicken, they were housed in the separate cage. According to the different therapeutic doses of antibiotics for poultry, each group of chickens was injected with the corresponding antibiotic or Phosphate Buffer Saline (NC was injected once a day for three consecutive days; LH was injected twice a day for three consecutive days; GS was injected twice a day for three consecutive days; FI was injected once every two days for a total of two injections; BP was injected twice a day for three consecutive days; CS was injected once a day for three consecutive days; EI was injected once a day for three consecutive days). Randomly collected three fecal samples from each antibiotic group in the morning on Days 0, 3, 7, 11.

**Table S1.** Antibiotic treatments in different groups.

| Groups | Manufacturers | Lot Number | Medication dosage | Frequency of injection |
| --- | --- | --- | --- | --- |
| Normal control (NC) | Phosphate Buffer Saline (PBS) | | 0.1 mL/bird | Once/day for 3 days |
| Lincomycin hydrochloride (LH) | HEFEI ZHONGLONGSHENLI ANIMAL PHARMACEUTCAL CO., LTD., HEFEI, CHINA | 20240713 | 0.03 mL/bird | Twice/day for 3 days |
| Gentamicin sulfate (GS) |  | 20240907 | 0.006 mL/bird | Twice/day for 3 days |
| Florfenicol injection (FI) |  | 20240816 | 0.006 mL/bird | Once/two days for 4 days |
| Benzylpenicillin potassium (BP) |  | 20240811 | 0.325 mL/bird | Twice/day for 3 days |
| Ceftiofur sodium (CS) |  | 20240905 | 0.1 mL/bird | Once/day for 3 days |
| Enrofloxacin infection (EI) | ANHUI QIANSHOU BIOTECHNOLOGY CO., LTD., HEFEI, CHINA | 24080301 | 0.01 mL/bird | Once/day for 3 days |

**Table S2.** Microbiota sequencing parameters of fecal samples

| **Groups** | **Sample ID** | **Input** | **Filtered** | **Denoised** | **Non-chimeric** | **Non-singleton** |
| --- | --- | --- | --- | --- | --- | --- |
|  | NC 0d 3_1 | 62839 | 53124 | 52574 | 47020 | 33354 |
| NC0 | NC 0d 3_2 | 60135 | 50910 | 50335 | 45202 | 32370 |
|  | NC 0d 3_3 | 59673 | 50425 | 49918 | 44704 | 31934 |
|  | NC 3d 3_1 | 60847 | 51296 | 50519 | 45788 | 34001 |
| NC3 | NC 3d 3_2 | 63733 | 53934 | 53154 | 46830 | 35800 |
|  | NC 3d 3_3 | 60398 | 51054 | 50317 | 44905 | 33531 |
|  | LH 3d 3_1 | 61390 | 51835 | 51163 | 45622 | 31186 |
| LH3 | LH 3d 3_2 | 61447 | 51666 | 50973 | 44899 | 30750 |
|  | LH 3d 3_3 | 64343 | 54212 | 53459 | 47388 | 31603 |
|  | GS 3d 3_1 | 63579 | 53579 | 52939 | 48771 | 25523 |
| GS3 | GS 3d 3_2 | 61181 | 51710 | 51065 | 47341 | 24768 |
|  | GS 3d 3_3 | 61928 | 52262 | 51542 | 48006 | 23929 |
|  | FI 3d 3_1 | 60313 | 50961 | 50450 | 44130 | 38278 |
| FI3 | FI 3d 3_2 | 61933 | 61933 | 61492 | 54769 | 50129 |
|  | FI 3d 3_3 | 62504 | 62504 | 61996 | 55435 | 50315 |
|  | BP 3d 3_1 | 64170 | 54295 | 53746 | 46305 | 38133 |
| BP3 | BP 3d 3_2 | 63194 | 53409 | 52841 | 45286 | 37748 |
|  | BP 3d 3_3 | 63477 | 53662 | 53178 | 45716 | 38728 |
|  | EI 3d 3_1 | 60609 | 51232 | 50770 | 43570 | 39406 |
| EI3 | EI 3d 3_2 | 64069 | 53927 | 53457 | 46111 | 41536 |
|  | EI 3d 3_3 | 65418 | 55208 | 54787 | 47527 | 43158 |
|  | CS 3d 3_1 | 60406 | 51015 | 50269 | 44496 | 33398 |
| CS3 | CS 3d 3_2 | 64117 | 54118 | 53340 | 46485 | 34166 |
|  | CS 3d 3_3 | 64308 | 54302 | 53511 | 46916 | 34210 |
|  | NC 7d 3_1 | 65229 | 55107 | 54450 | 49506 | 38394 |
| NC7 | NC 7d 3_2 | 65297 | 55081 | 54507 | 50097 | 40377 |
|  | NC 7d 3_3 | 65127 | 55008 | 54342 | 49058 | 38101 |
|  | LH 7d 3_1 | 62702 | 52920 | 52299 | 47946 | 28589 |
| LH7 | LH 7d 3_2 | 65326 | 55177 | 54530 | 49498 | 31868 |
|  | LH 7d 3_3 | 64013 | 54002 | 53371 | 48878 | 30677 |
|  | GS 7d 3_1 | 65092 | 54999 | 54505 | 48972 | 37832 |
| GS7 | GS 7d 3_2 | 61349 | 51654 | 51232 | 46256 | 35075 |
|  | GS 7d 3_3 | 65693 | 55589 | 55111 | 49708 | 37425 |
|  | FI 7d 3_1 | 60055 | 50751 | 50399 | 43792 | 39998 |
| FI7 | FI 7d 3_2 | 64061 | 54427 | 54009 | 46540 | 42455 |
|  | FI 7d 3_3 | 62143 | 52515 | 52089 | 44899 | 41289 |
|  | BP 7d 3_1 | 64410 | 54464 | 53837 | 47979 | 38790 |
| BP7 | BP 7d 3_2 | 63428 | 53428 | 52879 | 46965 | 38127 |
|  | BP 7d 3_3 | 60709 | 51265 | 50742 | 45354 | 37081 |
|  | EI 7d 3_1 | 64141 | 54126 | 53474 | 48241 | 30384 |
| EI7 | EI 7d 3_2 | 62148 | 52458 | 51834 | 46687 | 29111 |
|  | EI 7d 3_3 | 59363 | 50113 | 49465 | 44993 | 28126 |
|  | CS 7d 3_1 | 64972 | 54727 | 54053 | 49303 | 26732 |
| CS7 | CS 7d 3_2 | 61723 | 52101 | 51507 | 46636 | 25061 |
|  | CS 7d 3_3 | 63236 | 53367 | 52712 | 48251 | 24571 |
|  | NC 11d 3_1 | 59593 | 50072 | 49387 | 45001 | 37725 |
| NC11 | NC 11d 3_2 | 65950 | 55671 | 54928 | 49803 | 42306 |
|  | NC 11d 3_3 | 62101 | 52360 | 51756 | 47005 | 39793 |
|  | LH 11d 3_1 | 63141 | 53473 | 52520 | 40735 | 28064 |
| LH11 | LH 11d 3_2 | 59689 | 50601 | 49702 | 39075 | 26389 |
|  | LH 11d 3_3 | 63705 | 53895 | 52911 | 42005 | 29006 |
|  | GS 11d 3_1 | 59570 | 50408 | 49814 | 41985 | 28621 |
| GS11 | GS 11d 3_2 | 65074 | 55057 | 54380 | 46552 | 31349 |
|  | GS 11d 3_3 | 62777 | 53034 | 52360 | 44514 | 30381 |
|  | FI 11d 3_1 | 62751 | 53153 | 52359 | 39566 | 32913 |
| FI11 | FI 11d 3_2 | 62230 | 52478 | 51736 | 38831 | 32034 |
|  | FI 11d 3_3 | 60290 | 51118 | 50394 | 38881 | 31881 |
|  | BP 11d 3_1 | 60134 | 50740 | 49520 | 38688 | 29491 |
| BP11 | BP 11d 3_2 | 65583 | 55446 | 54087 | 42537 | 32355 |
|  | BP 11d 3_3 | 63650 | 53780 | 52468 | 40538 | 31156 |
|  | EI 11d 3_1 | 64967 | 54839 | 54105 | 48766 | 35350 |
| EI11 | EI 11d 3_2 | 62251 | 52463 | 51779 | 47387 | 34473 |
|  | EI 11d 3_3 | 63494 | 53768 | 53099 | 47265 | 33821 |
|  | CS 11d 3_1 | 63062 | 53149 | 52539 | 47304 | 34244 |
| CS11 | CS 11d 3_2 | 61432 | 51938 | 51262 | 46608 | 33711 |
|  | CS 11d 3_3 | 63742 | 53759 | 53165 | 48322 | 35314 |
| **Mean** | | **62748.7** | 53288.7 | 52627.5 | 46123.5 | 34218.1 |
| **Standard Error** | | **229.2** | 275.4 | 278.2 | 405.9 | 689.5 |
